# Intracoastal shipping drives patterns of regional population expansion by an invasive marine invertebrate

**DOI:** 10.1002/ece3.362

**Published:** 2012-09-13

**Authors:** John A Darling, Leif-Matthias Herborg, Ian C Davidson

**Affiliations:** 1 National Exposure Research Laboratory, United States Environmental Protection Agency109 TW Alexander Drive, Durham, North Carolina, 27711; 2 British Columbia Ministry of EnvironmentP.O. Box 9338 Stn Prov Govt, Victoria, British Columbia, V8W9M1, Canada; 3 Aquatic Bioinvasion Research and Policy Institute, Portland State University & Smithsonian Environmental Research CenterP.O. Box 751-ESR, Portland, Oregon, 97207-0751

**Keywords:** Invasive species, microsatellite, population genetics, *Styela clava*, vector analysis

## Abstract

Understanding the factors contributing to expansion of nonnative populations is a critical step toward accurate risk assessment and effective management of biological invasions. Nevertheless, few studies have attempted explicitly to test hypotheses regarding factors driving invasive spread by seeking correlations between patterns of vector movement and patterns of genetic connectivity. Herein, we describe such an attempt for the invasive tunicate *Styela clava* in the northeastern Pacific. We utilized microsatellite data to estimate gene flow between samples collected throughout the known range of *S. clava* in the region, and assessed correlation of these estimates with patterns of intracoastal commercial vessel traffic. Our results suggest that recent shipping patterns have contributed to the contemporary distribution of genetic variation. However, the analysis also indicates that other factors—including a complex invasion history and the influence of other vectors—have partially obscured genetic patterns associated with intracoastal population expansion.

## Introduction

The risks posed by invasive populations depend in large part on their ability to expand their nonnative ranges through colonization of new habitats. Many authors have recognized the importance of factors driving movement of invasive propagules (“propagule pressure”) not only to sites of initial colonization outside a taxon's native range, but also to sites of secondary colonization following establishment (Lockwood et al. 2005, 2009; Simberloff 2009). Uncovering the environmental parameters—including anthropogenic dispersal vectors—favoring expansion of invasive populations is thus a critical step toward informing accurate risk assessments and developing effective management practices and science-based policies (Estoup and Guillemaud 2010; Estoup et al. 2010).

Efforts to understand the role of various colonization processes in determining the pattern and extent of biological invasions have generally fallen into two categories. First, many attempts have been made to predict the spread of nonnative populations utilizing information on likely drivers of population expansion. In particular, researchers have focused on environmental factors captured in niche models (Chen et al. 2007; Therriault and Herborg 2008; Compton et al. 2010; Thum and Lennon 2010) and on the movement of specific anthropogenic vectors of introduction and spread (Herborg et al. 2007a, 2009; Kornis and Vander Zanden 2010). Second, population genetic approaches have frequently been utilized to infer invasion histories, including the most probable pathways of past population expansion (Hampton et al. 2004; Shoemaker et al. 2006; Roux et al. 2010).

Unfortunately, there has been little rigorous effort to merge quantitatively these prospective and retrospective approaches. For instance, models forecasting invasive spread based on vector movements (Leung et al. 2006; Bossenbroek et al. 2007) make specific predictions regarding the connectivity between nonnative populations, predictions that could be—but, to our knowledge, have not been—tested by genetic analyses. Similarly, population genetic reconstructions of invasion history are commonly accompanied by *post hoc* validation, typically involving recognition of potential invasion pathways and/or vectors likely to have contributed to the inferred colonization scenarios. However, in very few cases, has such validation involved explicit attempts to correlate quantitatively hypothesized explanatory factors with observed patterns of population genetic connectivity. One considerable obstacle to such efforts has been the lack of data that allow construction of truly directional, source-to-destination connectivity networks of anthropogenic vectors. Thus, even when quantitative assessments of correlation between vector intensity and genetic connectivity have been attempted, they have typically relied upon nondirectional measures of vector connectivity (e.g., Herborg et al. 2007b; Lacoursière-Roussel et al. 2012). Given the difficulties associated with direct measurement of propagule pressure (Simberloff 2009), studies that merge indirect genetic inference of population connectivity with predictions of colonization scenarios based on vector and/or environmental data should prove increasingly valuable.

**Figure 1:**
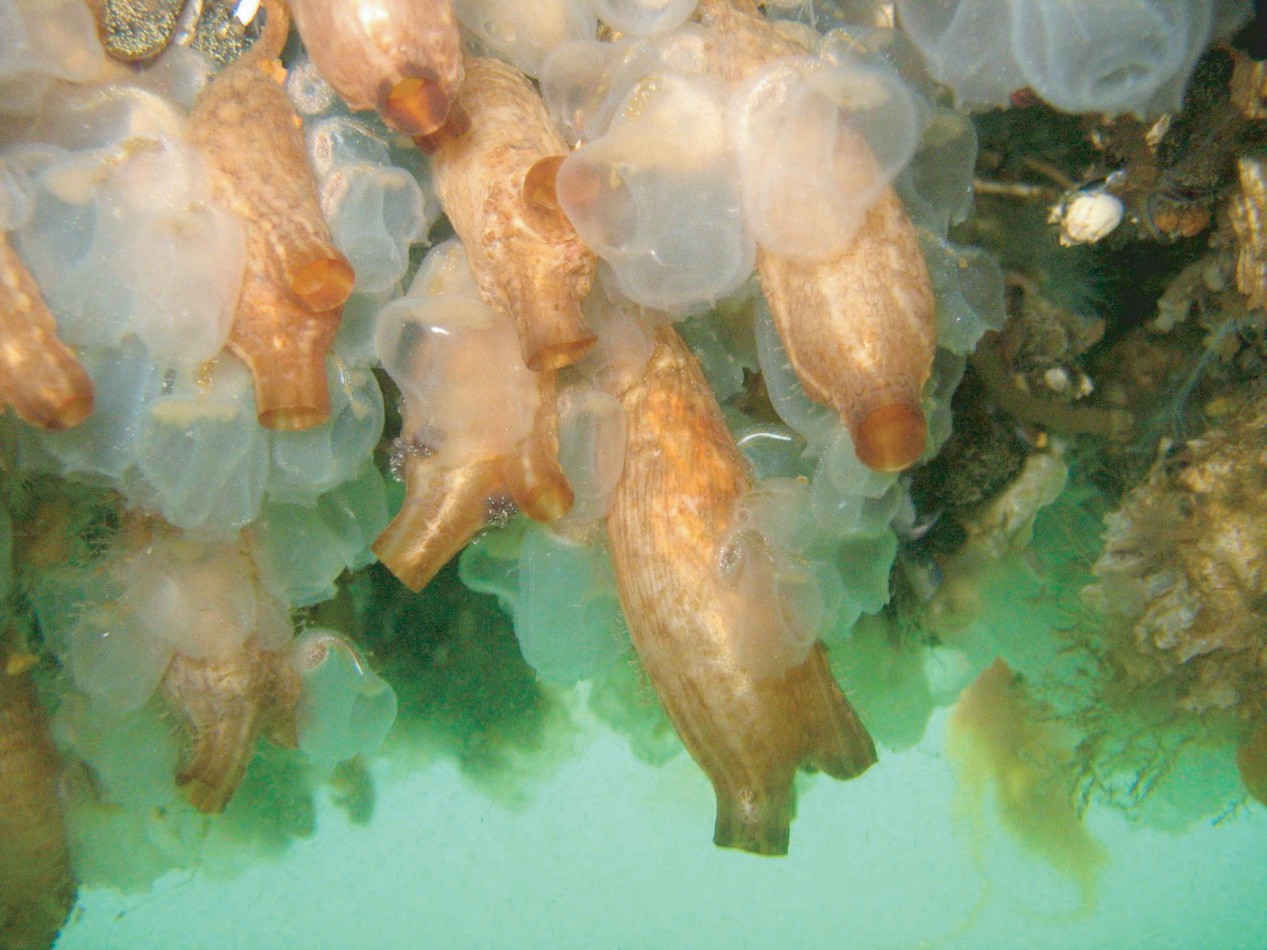
The invasive club tunicate *Styela clava* mixes with the native tunicate *Corella inflata* (clear tunic) in a dense fouling community on the underside of a floating dock at Pleasant Harbor Marina, Washington USA. Photo by Janna Nichols.

Herein, we describe the integration of population genetic and vector analyses to investigate the factors driving regional expansion of the invasive ascidian *Styela clava* in the northeastern Pacific. *S. clava* is a large solitary tunicate typically restricted to sheltered marine habitats, such as those associated with harbors and marinas (Lambert and Lambert 1998; Fig. 1). Although native to East Asia, the species has become a common resident of marine fouling communities globally. *Styela clava* is an oviparous hermaphrodite, producing planktonic eggs that develop into lecithotrophic larvae, which settle out of the water column within 24–48 h after spawning (Davis and Davis 2007; Dupont et al. 2009). Despite this restriction to natural dispersal capacity, *S. clava* has exhibited an impressive capacity for population spread at local and regional scales (Davis and Davis 2007). Recent studies have suggested that post establishment dispersal of propagules is likely driven to a large extent by human agency, most likely among fouling assemblages on slow-moving vessels (Dupont et al. 2009; Goldstien et al. 2010). In the eastern Pacific, *S. clava* was first reported in southern California in the early 1930s and subsequent spread has been dramatic, with populations establishing as far north as Vancouver Island, British Columbia by the late 1990s (Lambert and Lambert 1998). Given the substantial negative ecological and economic impacts of this species (Minchin et al. 2006a), more thorough understanding of the factors contributing to population expansion may prove critical to effective future management efforts.

In the current study, we employed 10 highly polymorphic nuclear microsatellite markers (Dupont et al. 2006) and data on the intracoastal movement of commercial vessels to test the hypothesis that patterns of recent commercial ship traffic have contributed to the expansion of *S. clava* in the region between San Diego, CA and Vancouver Island. Our analysis of vessel movements allows us to construct a true source-to-destination connectivity network, and thus offers a novel opportunity to quantitatively assess hypotheses regarding anthropogenic drivers of invasive spread. We tested for statistical strength of correlation between this vector network and both equilibrium and nonequilibrium estimates of genetic connectivity to determine whether or not vessel traffic shapes genetic structure in the study region.

## Materials and Methods

### Compilation of shipping data

**Figure 2:**
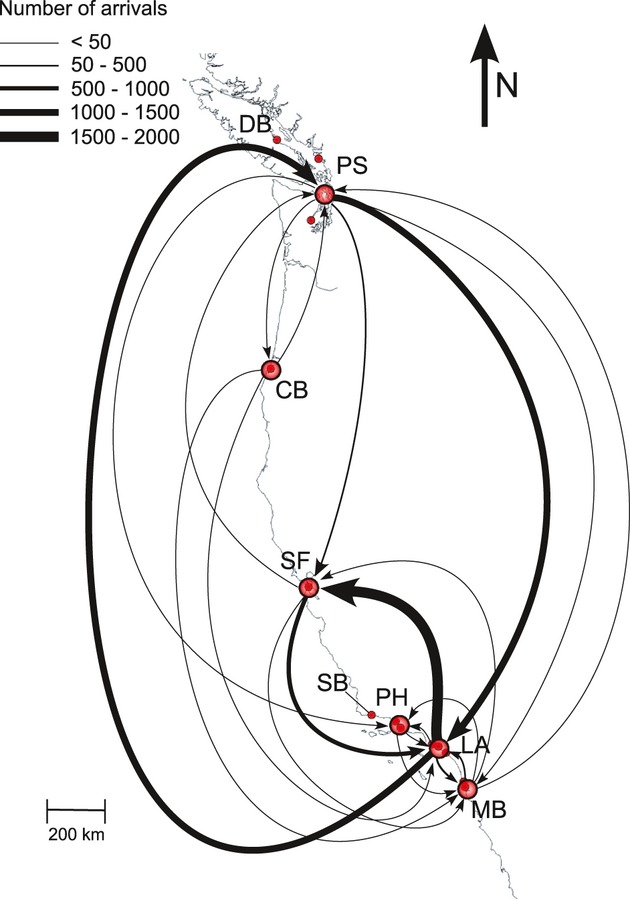
Hypothetical *Styela clava* dispersal network based on shipping connectivity matrix. Individual sampling sites for genetic data are indicated by small circles; larger circles indicate clustered sites following Table 1. Arrows indicating directional shipping connectivity between sites (see Table 1 for cluster labels) are weighted based on vector intensity as specified in the figure legend.

Commercial vessel arrivals for California, Oregon, Washington, and British Columbia were analyzed using data from each state's ballast water reporting program (US states) and the Canadian Marine Communications and Traffic Services database (BC). These data represented >96% of all vessel arrivals from a 2-year period (2003–2005) for US ports and for 1-year in BC. For all regions, data included arrival port, date-of-arrival, and previous port. The only suspected minor under-reporting of vessels may have occurred for barges because regulatory changes affecting barges occurred at the time. The database comprised more than 32,000 vessel arrivals, and patterns of connectivity among ports (and from overseas ports) were derived from a common denominator of one annual cycle of vessel flux (Fig. 2).

### Molecular methods

*Styela clava* were collected in 2006 as part of a large-scale effort to assess the distribution of genetic variation within multiple established and widely distributed coastal marine invasive species in the northeastern Pacific. Collections were conducted at a total of 36 sites along the Pacific US coast, including all known sites where *S. clava* populations had been previously reported. *Styela clava* specimens were successfully collected from eight of those sites (Table 1). Subsequent similar collection efforts in the Puget Sound region (Blaine Marina and Pleasant Harbor) and Vancouver Island (Deep Bay) yielded three additional samples, and *S. clava* DNA specimens extracted from animals previously collected at Santa Barbara Harbor were provided courtesy of J Bishop and L Dupont. In all collection efforts, whole animals were removed by scraping of individuals from artificial hard substrates, primarily pier pilings, docks, and marina floats. Specimens were stored in 95% ethanol prior to genetic analysis. Genomic DNA was extracted from tissues using DNeasy extraction kits (Qiagen, Valencia, California) following the manufacturer's protocol.

**Table 1:** Summary of *Styela clava* collections

Cluster	Site ID	Site name	Latitude	Longitude	*n*	*N*_A_	*A*_R_	*H*_E_	*F*_IS_
1. SD	SD	San Diego	32.7237	−117.2238	58	4.9	4.01	0.57	0.28
	MB	Mission Bay	32.7671	−117.2362	33	4.6	4.05	0.55	0.22
2. LA	DP	Dana Point	33.4622	−117.7063	31	4.0	3.58	0.54	0.18
	NB	Newport Bay	33.6194	−117.8933	40	3.8	3.38	0.53	0.25
	LA	Los Angeles	33.7446	−118.2762	31	4.5	4.03	0.59	0.25
3. PH	PH	Port Hueneme	34.1482	−119.2020	19	3.5	3.44	0.55	0.32
	CI	Channel Islands	34.1641	−119.2255	37	4.4	3.83	0.55	0.24
4. SB	SB	Santa Barbara	34.4053	−119.6919	38	4.8	4.06	0.53	0.36
5. SF	SF	San Francisco	37.7785	−122.2520	34	4.2	3.69	0.54	0.35
6. CB	CB	Coos Bay	43.3476	−124.3233	18	3.8	3.62	0.56	0.20
7. PS	PL	Pleasant Harbor	47.6624	−122.9175	50	4.4	3.68	0.54	0.21
	BM	Blaine Marina	48.9923	−122.7594	46	4.4	3.77	0.54	0.24
8. DB	DB	Deep Bay	49.4648	−124.7284	49	5.2	4.14	0.58	0.19

*n*, sample size; *N*_A_, number of alleles; *A*_R_, allelic richness; *H*_E_, expected heterozygosity, *F*_IS_, fixation index.

Polymerase chain reaction amplification of 10 species-specific microsatellite loci (H1, 1C8, 1A9, 2H9, 3G9, 2E10, 3E1, 2G5, 1D11, and 2B12) was conducted using the PCR cycling parameters described by Dupont et al. (2006). Reactions were conducted in 15 μL total volume containing 0.5 U Taq DNA polymerase (Qiagen), 1x PCR buffer, 1 μmol/L each forward and reverse primer, 1 mmol/L dNTPs, 1.6 mmol/L MgCl_2_, and 10–100 ng of template DNA. Amplified products were sized on an ABI 3730xl DNA Analyzer using GeneScan-500 LIZ size standard (ABI, Carlsbad, California) and raw data were analyzed using GENEMARKER v. 1.60 (SoftGenetics, LLC, State College, Pennsylvania).

### Genetic analysis

Population structure was assessed using individual-based Bayesian inference implemented in STRUCTURE v. 2.2 (Falush et al. 2003). To determine the number of clusters (*K*) best supported by the genetic data, we assessed models with *K* ranging from 1 to the total number of samples (13). For each value of *K*, we conducted five independent Markov–Chain Monte Carlo (MCMC) runs with 10^5^ generations discarded as burn-in followed by an additional 10^6^ generations of data collection. The value of *K* best representing the population structure present in the genetic data was inferred using the procedure described by Evanno et al. (2005). Genetic relationships between samples were also assessed by three-dimensional factorial correspondence analysis (FCA) as implemented in the software GENETIX v. 4.0.5 (Belkhir et al. 2004).

To assess genetic relatedness between sample clusters for hypothesis testing, we calculated pairwise *F*_ST_ using MSANALYZER (Dieringer and Schlötterer 2002). We also adopted a nonequilibrium method of assessing gene flow via migration between samples based on genetic assignment testing. For all individuals, we determined the most likely assignment to clusters using the Bayesian assignment criteria of Rannala and Mountain (1997) as implemented in GENECLASS v. 2.0 (Piry et al. 2004). Directional gene flow between two clusters was then estimated as the proportion of individuals from a recipient cluster that assigned with greatest likelihood to a particularsource cluster.

Descriptive statistics for each sample were calculated using FSTAT v. 2.9.3.2 (Goudet 2001). In the case of allelic richness, estimates were corrected for sample size by rarefaction to 18 individuals. Allele frequencies were calculated using MSANALYZER (Dieringer and Schlötterer 2002). Tests for population bottlenecks were conducted using the Wilcoxon test implemented in BOTTLENECK, assuming a two-phase mutation model with 10% multistep mutations (Cornuet and Luikart 1996).

### Hypothesis testing

Unfortunately, failure to locate established populations at all sites along with occasional limitations on access to ports prevented collection of genetic data directly associated with all port sites. In addition, shipping data could not be compiled for all collection sites used in the genetic analysis, as not all sites were directly associated with commercial ports. Thus, all hypothesis testing was conducted using a genetic dataset in which a number of sites were clustered based on both Bayesian inference of population structure and geographic proximity to ports for which shipping data could be compiled. These clusters included San Diego and Mission Bay (cluster 1, “San Diego”); Dana Point, Newport Bay, and Los Angeles (cluster 2, “Los Angeles”); Port Hueneme and Channel Islands (cluster 3, “Port Hueneme”); and Pleasant Harbor and Blaine Marina (cluster 7, “Puget Sound”) (see Table 1 for assignment of collection sites to all eight clusters, along with site abbreviations; Fig. 2 illustrates geographic distribution of clusters). In two cases, clustering resulted in the inclusion of geographically distant individuals within single clusters: Dana Point is separated from Los Angeles by approximately 60 km, and Blaine Marina from Pleasant Harbor by approximately 150 km. However, in all cases, clustering was consistent with population genetic structure predicted using STRUCTURE analysis. Furthermore, unlike other clusters, neither Blaine Marina nor Pleasant Harbor are in immediate proximity to the port for which shipping data were collected, but rather represent the closest sites from which we were able to collect *S. clava* for genetic analysis.

We tested hypotheses by assessing statistical strength of correlation between pairwise dissimilarity matrices. Matrices were generated for both *F*_ST_ and directional migration estimates based on individual assignment tests; for geographic distance between samples (alongshore distance calculated in ArcGIS); and for shipping connectivity based on directional vessel visits (see Table S1). We tested for correlations between matrices using Mantel tests as implemented in the software CADM (Legendre and Lapointe 2004), which converts all matrices to ranked distances to account for deviations from normality and to allow for comparison of asymmetrical matrices. Spearman correlations were calculated with 9999 permutations for all tests to assess statistical significance.

## Results

### Genetic analysis

**Figure 3:**
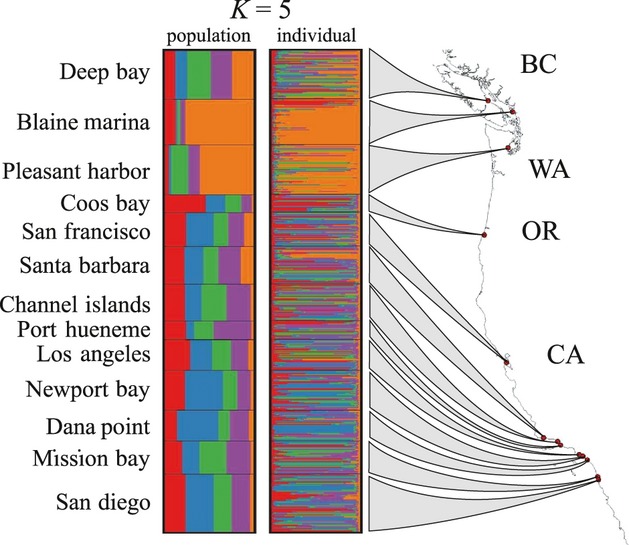
Population structure predicted using Bayesian clustering. Assignment of populations (left) and individuals (right) under model predicting *K* = 5 clusters. Each individual is represented by a thin horizontal line broken into *K* colored segments, with the length of each segment representing proportional membership of the individual in the cluster associated with that color. The run (out of 5 replicates) with the highest posterior probability is shown.

**Figure 4:**
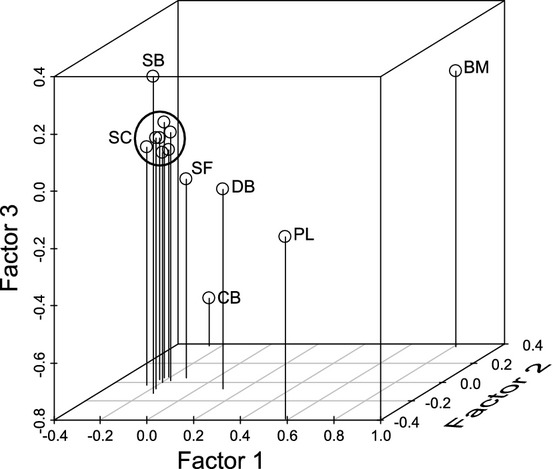
Factorial correspondence analysis (FCA). Sample ID's are as in Table 1, except for the circled cluster (SC), which includes all Southern California samples south of San Francisco except Santa Barbara.

**Figure 5:**
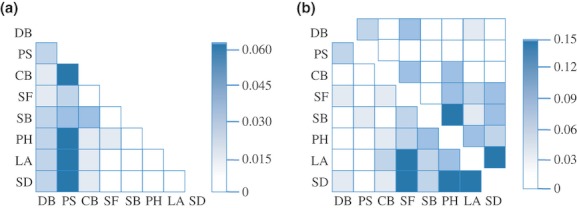
Pairwise *F*_ST_ genetic distance (a) and asymmetrical migration rates (b) between clusters (labels as in Table 1). For (b), rows represent source clusters and columns represent recipient clusters.

Bayesian inference suggested a model with *K* = 5 population clusters (Fig. 3). Plots of coancestry coefficients for all individuals revealed distinct clustering of individuals sampled from Blaine Marina and Pleasant Harbor (Puget Sound), which formed a single cluster highly differentiated from other collection sites. This was confirmed using Factorial Correspondence Analysis, which showed BM and PH as outliers to a large cluster comprising all other samples (Fig. 4). No significant genetic differentiation was observed between samples within the same cluster (not shown). Highest levels of genetic differentiation (*F*_ST_) were observed between the Puget Sound cluster and all other clusters, whereas migration rates were highest among southern clusters between San Francisco and San Diego (Fig. 5). Puget Sound also exhibited the highest proportion of private alleles, at 8.2%; most of these were relatively low frequency, although the most frequent represented 7.7% of the total diversity at one locus.

All samples showed significant heterozygosity deficits, with fixation indices similar to those observed elsewhere for *S. clava* (Goldstien et al. 2010). Genetic diversity measures were generally uniform across populations, with allelic richness (*A*_R_) ranging from 3.62 to 4.14 alleles and expected heterozygosity (*H*_E_) ranging from 0.53 to 0.59 (Table 1). No genetic bottlenecks were detected in any cluster using Wilcoxon test, and no cluster exhibited mode shifts in allele frequency away from the expected L-shaped distribution.

### Hypothesis testing

When considering the full dataset, the only significant correlation observed was that between *F*_ST_ and geographic distance (*R* = 0.6891, *P* = 0.0013; Table 2); *F*_ST_ was not significantly correlated with shipping intensity (*R* = −0.0406, *P* = 0.5743), nor was migration (*R* = 0.1723, *P* = 0.10). However, when Puget Sound was excluded from the dataset, the correlation between migration and shipping became significant (*R* = 0.5409, *P* = 0.0133). The correlation between *F*_ST_ and distance remained significant (*R* = 0.6429, *P* = 0.0129) in this case.

**Table 2:** Results of hypothesis testing. Correlations between matrices (*F*_ST_, geographic distance, gene flow via migration as estimated using assignments tests, and shipping intensity) were tested for the full dataset including Puget Sound (top) and a modified dataset excluding Puget Sound (bottom). Correlation coefficients (*R*) are shown above diagonal, *P* values below diagonal. Significant correlations are indicated in bold

	*F*_ST_	Distance	Migration	Shipping
*Including Puget Sound*				
F_ST_	–	**0.6891**	−0.6796	−0.0406
Distance	**0.0013**	–	−0.6136	−0.0293
Migration	0.9997	0.9989	–	0.1723
Shipping	0.5743	0.6198	0.2210	–
*Excluding Puget Sound*				
*F*_ST_	–	**0.6429**	−0.6465	−0.5189
Distance	**0.0129**	–	−0.5822	−0.3619
Migration	0.9983	0.9945	–	**0.5409**
Shipping	0.9694	0.8881	**0.0133**	–

## Discussion

Transport of *S. clava* as a component of fouling communities on maritime vessels, including commercial ships, is widely recognized as a prominent mechanism for both initial introduction and post establishment spread (Davis and Davis 2004, 2007; Minchin et al. 2006a). Our analyses indicate that patterns of recent commercial vessel traffic predict in part the distribution of contemporary genetic diversity observed among *S. clava* populations in the northeastern Pacific. Specifically, assessment of gene flow as determined using individual assignments tests is significantly correlated with shipping intensity (Table 2), supporting the hypothesis that commercial vessel traffic has been a significant driver of *S. clava* spread in the region.

This significant correlation was only observed when the cluster at Puget Sound (PS) was excluded from the analysis (Table 2). The PS samples (BM and PH) are genetically distinct from all other population clusters (Figs. 3, 4) and possessed a large percentage of private alleles (8.2% mostly low frequency alleles). Another recent study based on a smaller sample also recognized significant genetic differentiation between Puget Sound and other North American *S. clava* populations (Dupont et al. 2010). Although it remains uncertain what has led to this divergence between PS and other *S. clava* samples, one possibility is that the genetic differentiation of PS is due to an additional introduction, possibly from the native range, that was independent of the coastwide secondary spread of *S. clava* throughout the region. An independent influx of genetic diversity from outside the region would explain both the apparent genetic isolation of PS and the presence of private alleles in that cluster. The hypothesis of multiple introductions of *S. clava*—one in the 1920s to the LA area (Abbott and Johnson 1972) and one much more recently to the Puget Sound area—is consistent with analysis of international commercial vessel traffic, which revealed that LA/Long Beach and the Puget Sound region represent the two most common destinations for ships arriving to the North American west coast from the native range of *S. clava* (L.-M. Herborg and I. Davidson, unpubl. data). That hypothesis would also explain why the correlation between intracoastal shipping and distribution of genetic variation is only apparent when PS is excluded from the analysis, as the introduction of diversity from outside the region would obscure patterns driven by vectors within the region.

It is also possible that population bottlenecks combined with limited gene flow has led to substantial genetic drift and differentiation of the PS cluster. This would argue that, at least in this case, shipping intensity fails to reflect colonization and gene flow patterns, as PS is strongly connected via commercial shipping to other genetic clusters, most notably LA. However, as no such genetic bottlenecks were detected at PS, we believe this hypothesis to be less consistent with the observed data. A previous study similarly failed to detect founder effects among invasive *S. clava* populations in the eastern Pacific, including a population in Puget Sound (Dupont et al. 2010).

Moreover, interesting to note is that significant correlation between shipping and genetic connectivity was not observed when genetic distance was estimated using an equilibrium method (*F*_ST_). This failure of *F*_ST_ to capture the affect of shipping on colonization patterns may reflect violations of equilibrium assumptions. The expected inverse relationship between genetic distance as measured by *F*_ST_ and dispersal between populations assumes equilibrium between mutation and genetic drift (Rousset 1997). For invasive populations, the time since initial introduction and range expansion is in most cases insufficient for this equilibrium to have been achieved (Herborg et al. 2007b; Fitzpatrick et al. 2012). This will be particularly true of rapidly expanding populations with large effective sizes (Rieux et al. 2011). The probable violation of equilibrium assumptions, in the case of *S. clava*, is thus likely to render *F*_ST_ a poor estimator of recent gene flow among established populations. This may explain why our nonequilibrium estimate based on assignment testing appears to better reflect recent colonization patterns associated with commercial shipping.

There are several potential explanations for the relatively weak correlations observed between genetic and shipping patterns (*R* = 0.5409), even in the case, where that correlation is statistically significant. First, it is possible that the correlation is obscured by the activity of alternate dispersal vectors. There is reason to believe that at least two other vectors, recreational boating and the movement of aquaculture stocks, contribute substantially to the expansion of *S. clava* populations worldwide (Minchin and Duggan 1988; Minchin et al. 2006a,b; Locke et al. 2007). Both vectors have historically been highly active in the northeastern Pacific (Naylor et al. 2001; McKindsey et al. 2007; Davidson et al. 2010), and recent genetic studies have implicated recreational vessel movements in driving regional colonization patterns by *S. clava* in other parts of its nonnative range (Dupont et al. 2009; Goldstien et al. 2010). It is thus likely that other vectors have contributed to the spread of *S. clava* in the northeast Pacific, and possible that these vectors may be stronger predictors of the observed distribution of genetic variation. Unfortunately, data on the vessel flux and movement patterns for these vectors remain unavailable, at present, precluding direct tests of those relationships, such as we have performed herein for commercial vessels.

It is also possible that there may be temporal mismatch of the genetic and shipping datasets that would confound our analysis. *Styela clava* has been present on the US Pacific coast since at least the early 1930s (Lambert and Lambert 1998). In contrast, our vector data was collected for ship traffic in the years 2003–2005, and reflects modern patterns of vessel flux for our focal ports. While a comparison of the genetic structure with historical shipping data would have been desirable, such a long-term dataset with the resolution used in this study is not available. In general, shipping on the Pacific Coast of North America has grown dramatically over the 80-year period of *S. clava*'s residence in the region, and heavier traffic from faster and larger ships over time may well have played a role in redistributing *S. clava* northward. Unfortunately, data on directional patterns of historical vessel flux could not be readily assembled. The trend of apparent northward spread of invaders on the coast is well documented for many species and ships are strongly implicated (Ruiz et al. 2011), suggesting that modern vessel fluxes have played important roles in vectoring marine invasive species throughout the region. Still, unless contemporary genetic patterns reflect gene flow associated with these modern vessel fluxes, it is possible that our shipping dataset may fail to accurately capture the actual shipping patterns responsible for driving the distribution of genetic variation.

Our study demonstrates the feasibility of using genetic data to quantitatively test hypotheses regarding the mechanisms driving spread of invasive populations. In particular, it suggests that the combination of nonequilibrium genetic methods and vector datasets revealing true source-to-destination connectivity may provide a powerful means to assess correlations between anthropogenic drivers of dispersal and genetic structure. However, it also exposes difficulties likely to be associated with such attempts. First, the assembly of useful datasets on the movement of potential vectors may prove a limitation for many analyses. Previous studies focusing on aquatic bioinvasions have been forced to adopt various estimates of vector transport that are incapable of registering precise source-to-destination linkages between sites (Herborg et al. 2007a,b; Hoos et al. 2010). For example, Herborg et al. (2007b) calculated a “shipping volume index” as a proxy for ballast water exchange between European ports. Other studies have similarly used total ballast water discharge into ports as estimates of introduction effort (Herborg et al. 2007a). Elsewhere, Hoos et al. examined historical records of oyster plantings to estimate vector intensity for the Atlantic amethyst clam *Gemma gemma*. In none of these cases, was it possible to develop connectivity networks that quantify directional movement of vectors. The movements of recreational vessels have proven particularly challenging to assess given the lack of reporting requirements in most contexts, although researchers have developed various methods for overcoming the lack of empirical data (Padilla et al. 1996; Buchan and Padilla 1999; Leung et al. 2006; Bossenbroek et al. 2007). More recently, a study by Lacoursiere-Roussel et al. (2012) utilized interviews with recreational boaters to develop a connectivity network that was used to assess drivers of genetic structure among invasive *Botryllus schlosseri* populations in Nova Scotia. However, even that study was limited by an inability to register directionality of recreational boat traffic. Detailed databases that track directional vector intensities (e.g., the National Ballast Information Clearinghouse, http://invasions.si.edu/nbic; Verling et al. 2005) should improve researchers' ability to use genetic data to assess specific hypotheses regarding mechanisms of invasive spread.

Equally problematic is the fact that, for many invasive species, there will be multiple vectors potentially contributing to population expansion. Invasive spread is frequently determined by both natural and anthropogenic processes, and a large number of recent studies have revealed stratified dispersal patterns among invasive populations, with both local diffusive spread driven by natural dispersal mechanisms and regional spread driven by long-distance anthropogenic vectors (Wilson et al. 1999; Suarez et al. 2001; Muirhead et al. 2006; Shoemaker et al. 2006). For marine populations, in particular, multiple anthropogenic dispersal vectors may in fact be the norm (Fofonoff et al. 2003). These findings suggest that application of genetic data to reconstruct patterns of invasive spread will regularly need to unravel the effects of multiple drivers of that spread.

More generally, our results suggest that complex invasion histories, particularly those that involve multiple independent introductions, may make it problematic to infer the most important drivers of regional population expansion. By introducing external influences on contemporary distributions of genetic variation, multiple introductions will likely obscure the expected relationships between patterns of population genetic structure and patterns of regional vector intensity. Unfortunately, the past decade of invasion genetics research suggests that multiple introductions may be an all too common characteristic of successful biological invasions (Roman and Darling 2007; Dlugosch and Parker 2008). This is likely to be particularly true for species, such as *S. clava*, that continue to expand their global ranges through the activity of multiple anthropogenic dispersal vectors (Davis and Davis 2007; Locke and Carman 2009). A number of recent studies reveal that recognition of multiple introductions may force dramatic reconsideration of the most likely explanations for invasive spread, with corresponding implications for best management practices (Miller et al. 2005; Roman 2006). Advances in analytical approaches for reconstructing invasion histories based on genetic data should aid in recognition of multiple introduction scenarios (Estoup and Guillemaud 2010; Guillemaud et al. 2010), thus facilitating studies aimed at uncovering the critical environmental factors driving regional population expansion. Nevertheless, for many systems, disentangling the various mechanisms of invasive spread (e.g., natural dispersal, multiple anthropogenic vectors, and multiple introductions from external sources) will prove a challenging task.

## Supporting Information

**Table S1.** Distance matrices used in hypothesis testing.
