# Supplementary material for: Intracoastal shipping drives patterns of regional population expansion by an invasive marine invertebrate
Source: Ecol Evol. 2012 Sep 13;2(10):2557–66. doi: 10.1002/ece3.362 (PMC3492781; doi:10.1002/ece3.362)
Supplement: Supplementary file 1 [file ece30002-2557-SD1.docx]

Electronic Supplemental Material: Distance matrices used in hypothesis testing.

(*a*) *F*_ST_

|  | **DB** | **PS** | **CB** | **SF** | **SB** | **CI** | **LA** | **SD** |
| --- | --- | --- | --- | --- | --- | --- | --- | --- |
| **DB** | - |  |  |  |  |  |  |  |
| **PS** | 0.0370 | - |  |  |  |  |  |  |
| **CB** | 0.0306 | 0.0578 | - |  |  |  |  |  |
| **SF** | 0.0185 | 0.0390 | 0.0031 | - |  |  |  |  |
| **SB** | 0.0367 | 0.0456 | 0.0411 | 0.0082 | - |  |  |  |
| **PH** | 0.0352 | 0.0625 | 0.0208 | 0.0230 | 0.0129 | - |  |  |
| **LA** | 0.0380 | 0.0604 | 0.0223 | 0.0069 | 0.0103 | 0.0124 | - |  |
| **SD** | 0.0265 | 0.0531 | 0.0165 | 0.0055 | 0.0094 | 0.0098 | 0.0036 | - |

(*b*) Geographic distance

|  | **DB** | **PS** | **CB** | **SF** | **SB** | **CI** | **LA** | **SD** |
| --- | --- | --- | --- | --- | --- | --- | --- | --- |
| **DB** | - |  |  |  |  |  |  |  |
| **PS** | 919 | - |  |  |  |  |  |  |
| **CB** | 1106 | 1130 | - |  |  |  |  |  |
| **SF** | 2076 | 2100 | 972 | - |  |  |  |  |
| **SB** | 2774 | 2798 | 1670 | 743 | - |  |  |  |
| **PH** | 2836 | 2860 | 1732 | 805 | 62 | - |  |  |
| **LA** | 3017 | 3041 | 1913 | 986 | 243 | 181 | - |  |
| **SD** | 3164 | 3188 | 2060 | 1133 | 390 | 328 | 150 | - |

(c) Migration rates (assignment tests)

|  | **DB** | **PS** | **CB** | **SF** | **SB** | **CI** | **LA** | **SD** |
| --- | --- | --- | --- | --- | --- | --- | --- | --- |
| **DB** | - | 0.0816 | 0.0408 | 0.1224 | 0 | 0 | 0.0408 | 0.0204 |
| **PS** | 0.0833 | - | 0 | 0 | 0 | 0.0104 | 0.0104 | 0.0208 |
| **CB** | 0 | 0 | - | 0.1111 | 0 | 0.1111 | 0 | 0 |
| **SF** | 0.0588 | 0.0294 | 0.0588 | - | 0 | 0.1176 | 0.0882 | 0.1176 |
| **SB** | 0.0263 | 0.0526 | 0.0263 | 0.0789 | - | 0.1316 | 0.0526 | 0.1053 |
| **CI** | 0 | 0.0179 | 0.0536 | 0.0893 | 0.1071 | - | 0.1071 | 0.0714 |
| **LA** | 0.0098 | 0.0098 | 0.0784 | 0.1275 | 0.0882 | 0.1078 | - | 0.1373 |
| **SD** | 0.0549 | 0 | 0.0440 | 0.1429 | 0.0769 | 0.1319 | 0.1319 | - |

(*d*) Commercial shipping visits

|  | **DB** | **PS** | **CB** | **SF** | **SB** | **CI** | **LA** | **SD** |
| --- | --- | --- | --- | --- | --- | --- | --- | --- |
| **DB** | - | 0 | 0 | 0 | 0 | 0 | 0 | 0 |
| **PS** | 0 | - | 3 | 18 | 0 | 0 | 523 | 5 |
| **CB** | 0 | 18 | - | 0 | 0 | 0 | 0 | 0 |
| **SF** | 0 | 153 | 0 | - | 0 | 0 | 1944 | 21 |
| **SB** | 0 | 0 | 0 | 0 | - | 0 | 0 | 0 |
| **CI** | 0 | 16 | 0 | 0 | 0 | - | 28 | 34 |
| **LA** | 0 | 1065 | 13 | 550 | 0 | 36 | - | 78 |
| **SD** | 0 | 40 | 2 | 4 | 0 | 35 | 167 | - |
